# Overall Structural Alteration of Gut Microbiota and Relationships with Risk Factors in Patients with Metabolic Syndrome Treated with Inulin Alone and with Other Agents: An Open-Label Pilot Study

**DOI:** 10.1155/2022/2078520

**Published:** 2022-05-19

**Authors:** Ruiping Tian, Jiahui Hong, Jingjie Zhao, Dengyuan Zhou, Yangchen Liu, Zhenshan Jiao, Jian Song, Yu Zhang, Lingzhang Meng, Ming Yu

**Affiliations:** ^1^Department of Nutrition and Food Hygiene, School of Public Health, Tianjin Medical University, Tianjin, China; ^2^Life Science and Clinical Research Center, Affiliated Hospital of Youjiang Medical University for Nationalities, Baise, Guangxi Province, China; ^3^Department of General Family Medicine, Community Health Service Center of Zhongbei Town, Xiqing District, Tianjin, China; ^4^Department of Dermatology, Tianjin Academy of Traditional Chinese Medicine Affiliated Hospital, Tianjin, China; ^5^Center for Systemic Inflammation Research (CSIR), School of Preclinical Medicine, Youjiang Medical University for Nationalities, Baise, Guangxi Province, China

## Abstract

**Objective:**

The relative contribution of some products with prebiotic effects, such as inulin, together with medications specific to the human gut microbiome has not been comprehensively studied. The present study determined the potential for manipulating populations in the gut microbiome using inulin alone and combined with other agents in individuals with metabolic syndrome (MetS). The study also assessed whether there is relationship variability in multiple clinical parameters in response to intervention with the changes in the gut milieu. *Participants/Methods*. This single-centre, single-blinded, randomised community-based pilot trial randomly assigned 60 patients (mean age, 46.3 y and male, 43%) with MetS to receive either inulin, inulin+traditional Chinese medicine (TCM), or inulin+metformin for 6 months. Lipid profiles, blood glucose, and uric acid (UA) levels were analysed in venous blood samples collected after overnight fast of 8 h at baseline and at the end of the follow-up period. Microbiota from stool samples were taxonomically analysed using 16S RNA amplicon sequencing, and an integrative analysis was conducted on microbiome and responsiveness data at 6 months.

**Results:**

The results of 16S rRNA sequencing showed that inulin resulted in a higher proportion of Bacteroides at the endpoint compared with inulin+TCM and inulin+metformin (*p* = 0.024). More Romboutsia (*p* = 0.043), Streptococcus (*p* < 0.001), and Holdemanella (*p* = 0.011) were found in inulin+TCM and inulin+metformin samples. We further identified gut microbiota relationships with lipids, UA, and glucose that impact the development of MetS.

**Conclusion:**

Among the groups, inulin alone or combined with metformin or TCM altered specific gut microbiota taxa but not the general diversity. Accordingly, we analysed metabolites associated with microbiota that might provide more information about intrinsic differences. Consequently, a reliable method could be developed for treating metabolic syndrome in the future.

## 1. Introduction

Noncommunicable metabolic syndrome (MetS) is a growing public health concern worldwide. Metabolic syndrome is a constellation of metabolic disorders, characterised by abdominal adiposity, dyslipidaemia, low levels of high-density lipoprotein cholesterol (HDL. C), hypertension, and insulin resistance [1]. Metabolic syndrome directly affects health *via* the development of cardiovascular and cerebrovascular-related diseases and increases the risk of cancer [2]. While the oral administration of drugs can improve patient outcomes in terms of metabolic indexes, little is known about the status of the gut microbiota in such patients [3].

The human gastrointestinal tract houses hundreds of thousands of bacterial species [4]. Dysbiosis of the gut microbiota and/or structural alterations can trigger diseases and disrupt the epithelial barrier, which elicits an increase in the release of the endotoxin, lipopolysaccharide, from gram-negative bacterial cell walls into the systemic circulation, which triggers proinflammatory cytokine secretion [5]. Hence, many studies of gut microbes have vaulted to prominence [6]. Evidence from studies of humans and other animals has revealed a link between gut microorganisms and various components of MetS [7, 8]. Prebiotics are defined as substrates that are selectively utilised by host microorganisms and confer health benefits [9]. The ILSI Europe Prebiotic Expert Group and Prebiotic Task Force then proposed the concept of prebiotic effects defined as follows: the selective stimulation of growth and/or activity(ies) of one or a limited number of microbial genus(era)/species in the gut microbiota that confer(s) health benefits on the host [10]. Products with a prebiotic effect have thus been assessed in clinical trials in an attempt to improve gut microbial dysbiosis [10]. Moreover, the manipulation of gut microbiota *via* prebiotic interventions has provided evidence that gut microbial modulation helps to improve components or complications of metabolic syndrome [7].

Prebiotics favour the growth of beneficial bacteria, particularly those that produce short-chain fatty acids (SCFAs). Increased SCFAs in the intestine are associated with slowed weight gain, protection against systemic inflammation by increasing the gut barrier function, and improved glucose and lipid metabolism [11]. Inulin, a non-digestible dietary fibre, is a common prebiotic.

Traditional Chinese medicine (TCM) is a form of polypharmacy with a history of thousands of years. Natural medicines and their resulting bioactivities have been considered as therapeutic strategies against diseases [12], including MetS [13]. Some TCMs significantly affect glucose and lipid metabolism by regulating the gut microbiota, particularly bacteria that degrade mucin, have anti-inflammatory properties, produce lipopolysaccharides and SCFAs, or have bile-salt hydrolase activity [14]. The instant medicinal food packet in the present study consisted of *Coptis chinensis*, *Atractylodes macrocephala*, Tangerine peel, Coke malt, medicated leaves (stir-fried), and Hawthorn fruit (charred) according to a specific formula (Supplementary Table 1). *Coptis chinensis* (*Huang-Lian*), a common herb in TCM, is clinically effective in treating dyslipidaemia and hypercholesterolaemia [15]. Hawthorn has also been widely applied to manage hyperlipidaemia and cardiovascular diseases [13]. Medicated leaves improve human immunity through antioxidant and anti-inflammatory activities that lead to regulation of the gut microflora balance [16].

Metformin is linked to the composition of gut microbiota [17], even in healthy humans [18]. It is also applicable to the treatment or prevention of hyperlipidaemia and cardiovascular diseases [19].

The ability of prebiotic mix to regulate microbial communities in the host and benefit from symbiotic relationships among strains to improve their effects have been investigated [20]. Here, a prebiotic mix is simply a combination of products with prebiotic effects that benefit host health. However, to the best of our knowledge, little is known about the effects of inulin when combined with other agents on improving gut microcommunities in individuals with MetS. Overall, we postulated that the prebiotic mix would enhance microbial diversity in the host and take advantage of commensal relationships among organisms to confer more benefits on hosts.

The present study is primarily aimed at determining the influences of these products on gut microbiota structure and composition in patients with MetS. A more complete picture of the microbiota profile might provide greater clarity in terms of prioritising treatment for metabolic disorders and paving the way for further investigation.

## 2. Methods

### 2.1. Diagnostic Criteria for MetS

Clinical MetS was diagnosed when at least three of the following five conditions were met: waist circumference ≥ 90 or ≥80 cm in men and women, respectively; blood pressure, 120/80–140/90 mmHg; triglycerides, 150–200 mg/dL; HDL, <40 or <50 mg/dL in men and women, respectively, and fasting glucose 100–125 mg/dL.

### 2.2. Study Population

Residents of Tianjin for >5 years and aged 35–65 years were included if they had no inflammatory gastrointestinal disease or a history of gastrointestinal surgery within 5 years and had not been prescribed with antibiotics or other medications (proton pump inhibitors and H_2_ receptor antagonists) or dietary supplements (probiotics and prebiotics) or antacids that could affect the gut microbiota within 6 weeks before recruitment. They were provided with a pamphlet about the study during a physical examination and asked to participate in if eligible according to the above criteria. Written informed consent to participate was obtained from eligible 60 individuals who indicated an interest and met the inclusion criteria.

### 2.3. Intervention

All participants were randomly and equally assigned to take oral inulin, inulin+TCM formula, and inulin+metformin (*n* = 20 per group) for 6 months. They were instructed not to change their lifestyle, diet, and usual physical activities during the study and were contacted twice each month for 6 months by telephone or in-home visits to monitor side effects and compliance. None of the participants complained of adverse gastrointestinal reactions. All participants returned empty or remaining allocated packets to verify compliance at the endpoint.

### 2.4. Data Collection

Information about demographics and physical activity was collected from questionnaires. Physical activity was assessed based on whether the participants exercised daily. Body weight, height, waist circumference, and blood pressure were measured by on-site nurses. Height and weight were measured using a calibrated height–weight meter. Waist circumference was measured at the level of 0.5–1 cm above the navel using a tape measure. Mean blood pressure was calculated from three reads on the same arm after 5 min of rest in a semiupright position. Fasting plasma samples were also collected at baseline and at the end of the study. Serum glucose, total triglycerides (TGs), high-density lipoprotein (HDL), low-density lipoprotein (LDL), total cholesterol (T-CHOL), and uric acid (UA) were measured using an automated analyser.

Stool samples were collected at the end of the intervention period. The participants were provided with a stool collection kit with instructions about the proper way to collect stool samples. To represent the whole bacterial components/structure, the stools were homogenized vigorously, and one tablespoon of faecal samples was taken and placed in a labelled sterile conical tube, placed in a biohazard bag, delivered to the laboratory on ice, and stored at -80°C.

### 2.5. Metagenomic Measures

#### 2.5.1. Extraction of Genomic DNA

The concentration and purity of total bacterial DNA extracted from stool samples using the CTAB/SDS method (at Novogene Bioinformatics Technology Co. Ltd., Beijing, China) were monitored by electrophoresis on 1% agarose gels. Samples of DNA were then diluted to 1 ng/*μ*L in sterile water.

#### 2.5.2. Amplicon Generation and Purification

Bacterial genomic DNA was amplified using the specific primers 515F (GTGCCAGCMGCCGCGGTAA) and 806R (GGACTACHVGGGTWTCTAAT) for the V4 hypervariable regions of the 16S rRNA gene. All polymerase chain reactions (PCR) proceeded in 30 *μ*L volumes containing 15 *μ*L of Phusion® High-Fidelity PCR Master Mix (New England Biolabs Inc., Ipswich, MA, USA), 0.2 *μ*M forward and reverse primers, and approximately 10 ng of template DNA. The cycling conditions comprised 98°C for 1 min, followed by 30 cycles of 98°C for 10 s, 50°C for 30 s, 72°C for 30 s, and followed by 72°C for 5 min. The PCR products in an equal volume of 1X loading buffer (containing SYB green) were resolved by 2% agarose gel electrophoresis. Samples with a bright main band at 400–450 bp were mixed at an equal density and purified using Gene JET Gel Extraction Kit (Thermo Fisher Scientific Inc., Waltham, MA, USA).

#### 2.5.3. Metagenomic Sequencing and Analysis

Sequencing libraries were generated using Illumina TruSeq DNA PCR-Free Library Preparation Kits (Illumina Inc., San Diego, CA, USA) as described by the manufacturer, and index codes were added. The quality of the library was assessed using a Qubit@ 2.0 Fluorometer (Thermo Fisher Scientific Inc.) and an Agilent Bioanalyzer 2100 system. The library was sequenced on an Illumina NovaSeq platform, and 250 bp paired-end reads were generated. The raw 16S rRNA gene sequence reads were quality-filtered, merged, and clustered into operational taxonomic units (OTUs) with ≥97% similarity, and chimeric sequences were identified and removed. Representative sequences of each OTU were screened for further annotation. The sequencing quality of each sample including the purity of DNA was shown in supplementary table 2.

### 2.6. In Vitro Assay

Microplate assay and agar well diffusion assay were performed to test the relationship between herbal formula and specific bacteria.

#### 2.6.1. Microplate Assay

Inoculum containing 1% Romboutsia, Streptococcus, or Holdemanella was placed in LB medium (Invitrogen, #10855001), in flat-bottom 96-well plates. Meanwhile, equal volume of inulin, inulin+TCM, or inulin+metformin was added into the wells. The growth kinetics was monitored by microplate reader (Tecan Infinite M200) every two hours. Two wavelengths (700 nm and 520 nm) were used to avoid/minimize interference with background signals.

#### 2.6.2. Agar Well Diffusion Assay

Cream containing inulin, inulin+TCM, or inulin+metformin was, respectively, placed in 6 mm holes on Mueller Hinton Agar plates (ThermalFisher, #R01620), and after inoculating Romboutsia, Streptococcus, or Holdemanella, the plates were incubated at 35°C overnight. Diameters of the clear zones around the holes were measured and compared.

### 2.7. Statistical Analysis

All data were statistically analysed using the SPSS software (version 26.0; IBM, Armonk, NY, USA) and are expressed as means ± SD. Continuous and categorical variables for baseline characteristics were analysed using *t*-tests and *χ*^2^ tests, respectively. Gut microbiota were profiled on an intent-to-treat basis, regardless of whether the participants complied or completed the study. Sequences were analysed using the R software (version 3.5.2). Differences among groups were analysed using the Kruskal–Wallis chi-square test. All values with *p* < 0.05 were deemed significant.

## 3. Results

### 3.1. Participant Characteristics

The baseline characteristics of the 60 participants were similar (Table 1). At the end of the intervention, all participants were included in the analysis.

### 3.2. Changes in Gut Microbiota

**Figure 1:**
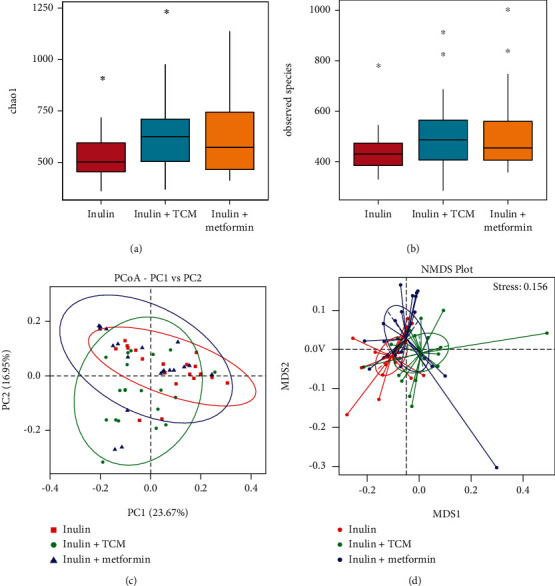
Shifts in enteric bacterial composition after 6 months of intervention. (a, b) Numbers of species and Chao1 diversity indices of intestinal bacteria assessed by 16S rRNA high-throughput sequencing significant differ (Kruskal–Wallis *χ*^2^ test; *n* = 20 per group). (c, d) PCoA and NMDS analyses of intestinal bacterial composition profiles. NMDS: nonmetric multidimensional; PCoA: principal coordinate analysis.

**Figure 2:**
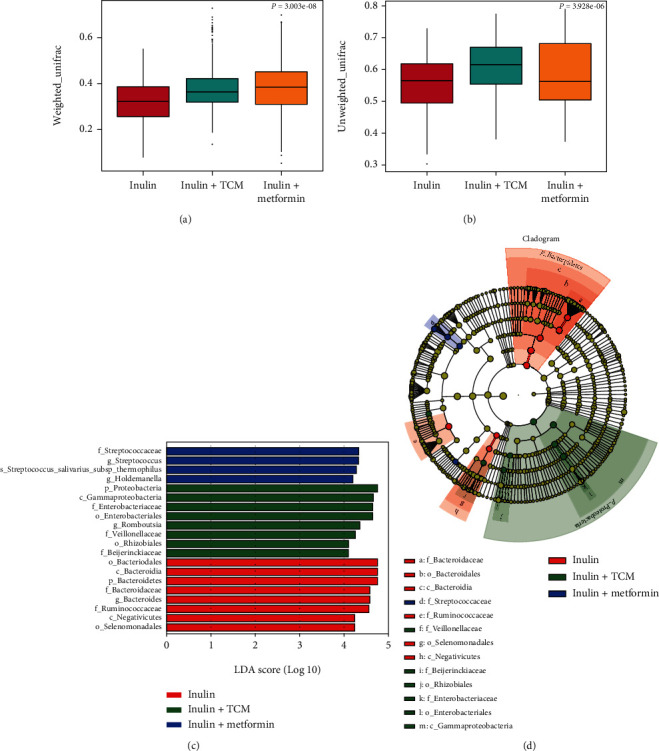
Comparison of *β* diversity among intestinal bacteria using weighted/unweighted UniFrac and LEfSe analyses after 6 months of intervention. (a, b) Weighted/unweighted UniFrac analysis of *β* diversity shows significant differences among intestinal bacteria (*n* = 20 per group; Kruskal–Wallis *χ*^2^ test). (c, d) Results of LEfSe assays show differences in gut bacteria abundance among groups and effect size of each differentially abundant bacterial taxa (*n* = 20 per group; significantly different, Wilcoxon rank sum tests).

Among 5,382,635 usable sequences obtained from all samples using the Illumina NovaSeq platform, 3,899,784 were high-quality, yielding an average of 64,996 sequences per sample. The results of the OTU analysis showed that the numbers of bacterial species did not change among the inulin, inulin+TCM, and inulin+metformin groups (*p* = 0.133 and *p* = 0.261, respectively; Figures 1(a) and 1(b)). However, weighted principal coordinate (PCoA), nonmetric multidimensional scaling (NMDS) (Figures 1(c) and 1(d)), and weighted/unweighted UniFrac analyses of the bacterial taxa among the three cohorts revealed significant differences in the gut microbiota composition among the groups (*p* = 3.003*e* − 08 and *p* = 3.928*e* − 06; Figures 2(a) and 2(b)). Analysis of group similarities (ANOSIM) also indicated significant differences among the three groups (*R* = 0.061, *p* = 0.013, Supplementary Figure 1).

**Figure 3:**
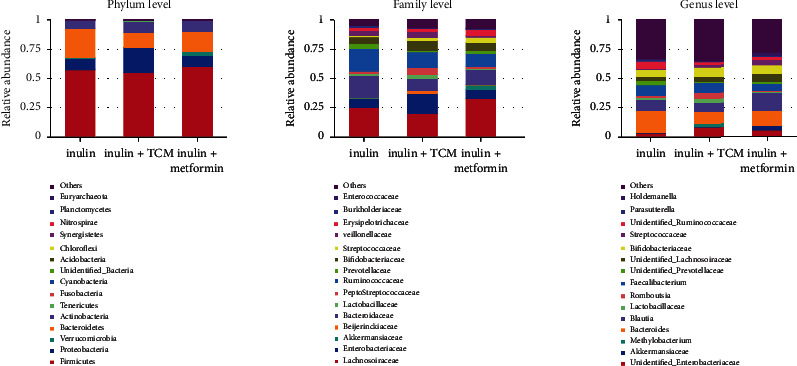
Relative abundance of bacterial taxa in the top 15 at phylum, family, and genus levels in patients with MetS treated with inulin, inulin+TCM, or inulin+metformin (*n* = 20 each) for 6 months. MetS: metabolic syndrome; TCM: traditional Chinese medicine.

To further confirm manipulation of the gut microbial community in the three groups, predominant bacterial species at the phylum, family, and genus levels were investigated by sequencing and analysing 16S rRNA.  Figure 3 shows bar plots of relative abundance at these levels. Consistent with previous findings, Bacteroidetes, Firmicutes, and Proteobacteria were the predominant phyla, followed by Actinobacteria. The abundance of Bacteroidetes at the phylum level was relatively increased (*p* < 0.05) in the inulin than in the other two groups (24.6% *vs.* 12.4% and 16.7%, respectively), whereas that of Proteobacteria (*p* < 0.05) in the inulin and TCM-treatment arms was 10.1%, 21.2%, and 9.7%, respectively. Firmicutes bacteria did not significantly differ (*p* > 0.05). Bacteroidaceae (*p* = 0.024) and Ruminococcaceae (*p* = 0.017) were the most abundant families in the inulin group, whereas Enterobacteriaceae (*p* = 0.017) and Veillonellaceae (*p* = 0.013) were the most abundant in the inulin TCM group, and Streptococcaceae (*p* < 0.001) was the most abundant in the inulin+metformin group. We analysed the top 15 genera to further evaluate modulation of the microbial community at the genus level. The relative proportion of Bacteroides was higher (*p* = 0.024) in the inulin than in the other two groups, but the abundance of Romboutsia (*p* = 0.043), Streptococcus (*p* < 0.001), and Holdemanella (*p* = 0.011) was greater in inulin+TCM and inulin+metformin samples, respectively.

The differential abundance among the groups was assessed using linear discriminant analysis Effect Size (LEfSe) assays (LDA > 4) (Figures 2(c) and 2(d)). The gut microbiota differed among the groups; Bacteroidetes (phylum), Bacteroidaceae and Ruminococcaceae (families), and Bacteroides (genus) were more abundant in the inulin group. Proteobacteria (phylum), Enterobacteriaceae and Veillonellaceae (families), and Romboutsia (genus) were dominant in the inulin+TCM group. Streptococcaceae (family) and Streptococcus and Holdemanella (genera) were significantly elevated in the inulin+metformin group.

Collectively, these results show similar numbers but significant differences in the types of bacteria in the gut microbiota among the three groups.

### 3.3. Correlations between Bacterial Abundance and MetS Risk Factors

**Figure 4:**
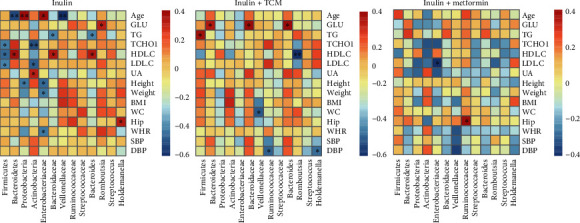
Heatmaps of correlations between species abundance (columns) and clinical parameters (rows) in patients with metabolic syndrome. Red and blue shades: positive and negative correlations, respectively. BMI: body mass index; DBP: diastolic blood pressure; GLU: glucose; HDL. C: high-density lipoprotein cholesterol; Hip: hip circumference; LDL. C: low-density lipoprotein cholesterol; SBP: systolic blood pressure; TCHO1: total cholesterol; TG: triglyceride; UA: uric acid; WC: waist circumference; WHR: waist to hip ratio (circumference). ^∗^Statistically significant correlations.

Correlations between the abundance and presence of different bacteria and the clinical parameters of the participants in each group were analysed to identify associations between host responsiveness and bacterial abundance.  Figure 4 summarises the results, which are detailed in the Supplementary Figure 1.

Several associations were significant in the inulin group (Figure 4).

Among the bacterial abundance that significantly and positively correlated with clinical parameters, HDL levels were closely associated with the abundance of *Bacteroidetes* (*r* = 0.522, *p* = 0.018), Bacteroidaceae (*r* = 0.485, *p* = 0.03), and *Bacteroides* (*r* = 0.485, *p* = 0.03). Some bacteria negatively correlated with the clinical parameters in the inulin group. For example, the abundance of *Actinobacteria* correlated negatively with T-CHOL (*r* = −0.594, *p* = 0.005) and LDL (*r* = −0.554, *p* = 0.011), whereas UA correlated positively (*r* = 0.457, *p* = 0.043). Similarly, the *Firmicutes* abundance significantly and negatively correlated with HDL (*r* = −0.522, *p* = 0.018).

Figure 4 shows the correlations between bacterial abundance and clinical parameters in the inulin+TCM group. Correlations between Romboutsia and HDL and between Veillonellaceae and WC were negative (*r* = −0.644, *p* = 0.002 and *r* = −0.505, *p* = 0.023, respectively).

Fewer significant correlations were found between bacterial abundance and clinical parameters in patients with MetS in the inulin+metformin than in the other two groups (Figure 4).

## 4. Discussion

This prospective study analysed the characteristics of the gut microbiota and their associations with risk factors in patients with MetS treated with various formulations. Little is known about these characteristics and correlations; therefore, the specific bacteria produced and the effects of interventions on the microbiota need to be understood to develop a basis for further investigation of optimal treatments for MetS.

The LEfSe results showed that the Ruminococcaceae family and the Bacteroides genus were more abundant in the inulin group. The Ruminococcaceae family plays an important role in dietary fibre degradation and is involved in the production of SCFAs that provide energy for the colonic epithelium and systemic nutrients [21]. SCFAs affect glucose homeostasis, lipid metabolism, regulation of the immune system, and the inflammatory response [22]. However, we did not identify associations between SCFAs and MetS traits, which might be partly due to a dietary imbalance.

The genus Bacteroides comprises gram-negative, obligate anaerobic, nonmotile, nonspore forming rods that are among the most prevalent components of the human intestinal microbiota and important degraders of polysaccharides in the human intestine [23]. Microorganisms in the colon can decompose “resistant” polysaccharides that are not metabolised during transit through the small intestine. Polysaccharide derivatives with an appropriately modified structure can improve the immunological activity of polysaccharides. They can actively enhance immune cells and regulate the immune function [24]. For example, sulfated polysaccharides can improve the role of polysaccharides in macrophage phagocytosis and promote the secretion of IL-6, IL-1*β*, and other interleukins by macrophages [25].

Higher proportions of the Enterobacteriaceae and Veillonellaceae families and the Romboutsia genus were associated with inulin+TCM. Some diseases are associated with a significantly higher abundance of Enterobacteriaceae and Veillonellaceae [26, 27]. Furthermore, both taxa were typically found together [26]. An increase in Enterobacteriaceae in gut-associated microbial populations is a microbial signature of epithelial dysfunction [28]. The abundance of Veillonellaceae is inversely associated with changes in the glucose response and IL-6 levels after prebiotic intake [29]. Romboutsia, an obesity biomarker, correlates positively and significantly with indicators of body weight (including waistline and body mass index), serum lipids (LDL, TGs, and T-CHOL), and UA in humans [30]. The genus Romboutsia also correlates positively with HDL in rodent models [31].

Streptococcus and Holdemanella genera were relatively more abundant in the inulin+metformin group. Streptococcus might increase levels of folate production and serum folate concentrations *via* the upregulation of folate-mediated one-carbon metabolism and fatty acid oxidation pathways. This would result in rapid and dramatic reductions in liver fat and other cardiometabolic risk factors [32]. However, Streptococcus has been linked to the development of multiple metabolic disorders, including atherosclerotic cardiovascular disease [33, 34]. A relationship between Holdemanella and sex-specific fat distribution has been identified. The Holdemanella genus is associated positively and negatively with android fat ratios in males and females, respectively [35]. Metformin is responsible for most of the gut microbiota changes and metabolic improvements linked to prebiotic intervention [36].

This study has some limitations. The magnitude of the effects of the interventions remains uncertain because the study cohort was extremely small to rule out the possibility of chance findings. A wash-out period was not applied, and therefore, previous gut bacterial features might have impacted outcomes. Larger studies are needed to confirm structural alterations after discrepant interventions. Changes could be determined in experimental animals after controlling for the environmental system. In terms of taxa, we did not obtain more information about the bacterial species that are more closely associated with physiological roles. Interplay might occur between diet/lifestyle and the gut microbiome and among microbiomes. The association between gut bacteria and corresponding metabolites was not analysed.

Interestingly, our *in vitro* study showed that density of Romboutsia, Streptococcus, and Holdemanella was not altered by the herbal formula used in this study, by microplate assay and agar well diffusion (supplementary figure 2). This indicated the herbal needs to be digested, either the intermediate products or by-products induced the alteration of gut microbiota. It would be necessary to clarify the exact product(s) in future study.

In summary, information is insufficient to conclude the effects of distinct interventions on MetS risk factors. Whether ingredients in the prebiotic mix act alone or in combination to modulate the gut microbiome remains unclear. Further studies are needed to be conducted to clarify the molecular mechanism that determine the effects of the prebiotic mix on the human gut microbiome. However, metabolites derived from microbiota might facilitate better characterisation of the relationship between microbiota and risk for MetS. The metabolic benefits associated with microbial alterations requires further investigation, as they will provide insight into the roles of microbial metabolites as potential candidate biomarkers in individuals with metabolic syndrome.

## Figures and Tables

**Table 1:** Baseline characteristics of participants (*n* = 20 per group).

	Inulin	Inulin+TCM	Inulin+metformin
Age (y)	42.9 ± 13.6	48.5 ± 11.6	47.4 ± 12.1
Male sex (*n* %)	8 (40)	8 (40)	10 (50)
BW (kg)	75.2 ± 13.4	71.8 ± 12.8	71.9 ± 6.8
BMI (kg m^−2^)	26.3 ± 2.4	25.8 ± 2.9	26.4 ± 2.4
WC (cm)	91.4 ± 10.2	90.7 ± 8.0	92 ± 5.7
SBP (mmHg)	123.8 ± 10.2	126.7 ± 9.7	123.1 ± 9.8
DBP (mmHg)	86.2 ± 13.4	80.2 ± 6.4	78 ± 6.8
GLU (mmol ^L-1^)	5.4 ± 0.5	5.8 ± 1.3	5.5 ± 0.6
TG (mmol ^L-1^)	1.2 (0.6)	1.65 (1.19)	1.35 (0.61)
T-CHOL (mmol ^L-1^)	4.53 ± 0.8	4.9 ± 1.1	4.47 ± 0.8
HDL (mmol ^L-1^)	1.3 ± 0.3	1.3 ± 0.4	1.2 ± 0.3
LDL (mmol ^L-1^)	3.2 ± 0.8	3.3 ± 0.9	3.2 ± 0.6
UA (mmol ^L-1^)	326.3 ± 86.1	314.5 ± 87.7	317 ± 74.2
PA (yes no.)	15	15	15

## Data Availability

The raw sequencing data and code generated or analysed in this study are available from the corresponding author upon reasonable request.

## Supplementary Materials

Supplementary 1 Supplementary Table 1: the composition of herbal formula. The table described the composition of the herbal formula, including the quantity information of each component.

Supplementary 2 Supplementary Table 2: the sequencing quality of each sample. The sequencing quality of each sample was tested and was provided for further conformation.

Supplementary 3 Supplementary Figure 1: analysis of similarities (ANOSIM) indicated that the difference among groups was significant (*R* = 0.061, *p* = 0.013). The violin plots showed the similarity analysis of gut microbiota among different groups.

Supplementary 4 Supplementary Figure 2: *In vitro* assay exhibited that specific bacterium was not altered. (A) Microplate assay showed that Romboutsia, Streptococcus, and Holdemanella were not altered by different treatments. Data represents similar results acquired from 5 repeated experiments. NS: no significant. Each group contained 8 samples. (B) Agar well diffusion assay showed Romboutsia, Streptococcus, and Holdemanella were not altered by different treatments. Data represents similar results acquired from 5 repeated experiments. NS: no significant. Each group contained 8 samples.
