# Supplementary material for: Overall Structural Alteration of Gut Microbiota and Relationships with Risk Factors in Patients with Metabolic Syndrome Treated with Inulin Alone and with Other Agents: An Open-Label Pilot Study
Source: Mediators Inflamm. 2022 May 19;2022:2078520. doi: 10.1155/2022/2078520 (PMC9136633; doi:10.1155/2022/2078520)
Supplement: Supplementary 1 — Supplementary Table 1: the composition of herbal formula. The table described the composition of the herbal formula, including the quantity information of each component. [file 2078520.f1.pdf]

Supplementary Table 1. The composition of herbal formula

Herbs	Boil-free granules weight (g/day) [a]
Atractylodes Macrocephala	15
Tangerine Peel	15
Coptis Chinensis	15
Coke Malt	15
Medicated Leaven(stir-fried)	15
Nawthorn Fruit(charred)	15

[a] Each herb was processed into boil-free granules and packed into individual bags.
