# Supplementary figures and images for: Overall Structural Alteration of Gut Microbiota and Relationships with Risk Factors in Patients with Metabolic Syndrome Treated with Inulin Alone and with Other Agents: An Open-Label Pilot Study
Source: Mediators Inflamm. 2022 May 19;2022:2078520. doi: 10.1155/2022/2078520 (PMC9136633; doi:10.1155/2022/2078520)

Supplementary Figure 1

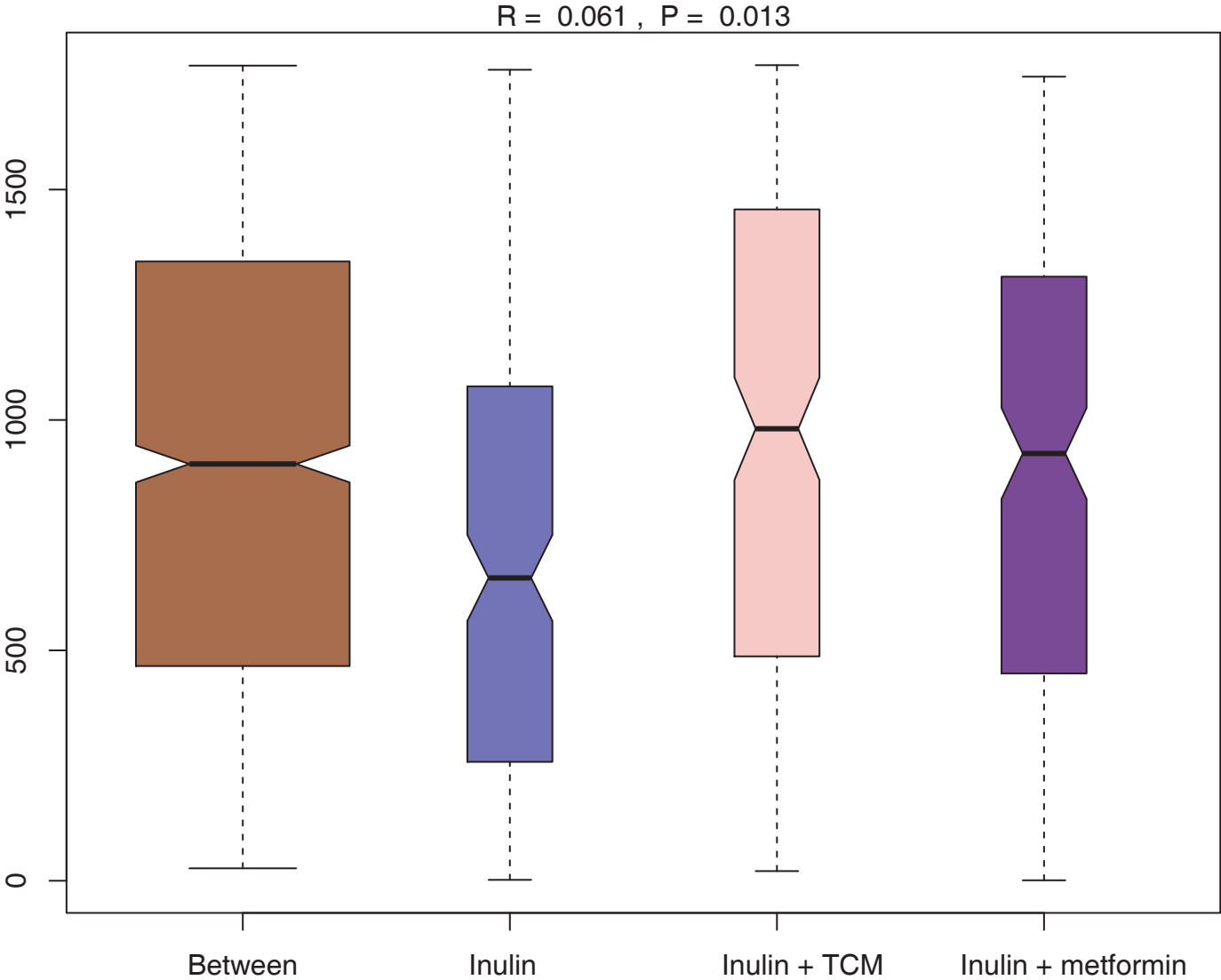

Supplement: Supplementary 3 — Supplementary Figure 1: analysis of similarities (ANOSIM) indicated that the difference among groups was significant (R = 0.061, p = 0.013). The violin plots showed the similarity analysis of gut microbiota among different groups. [file 2078520.f3.pdf]

Supplementary Figure 2

A

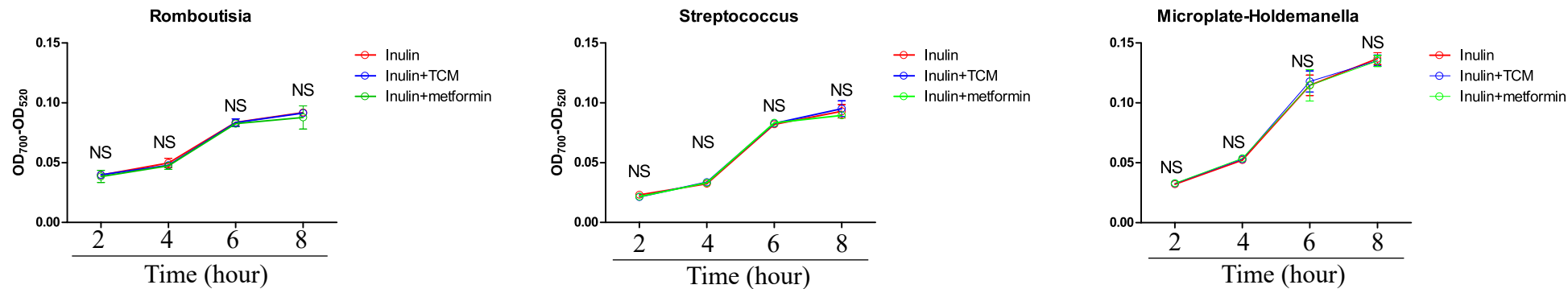

B

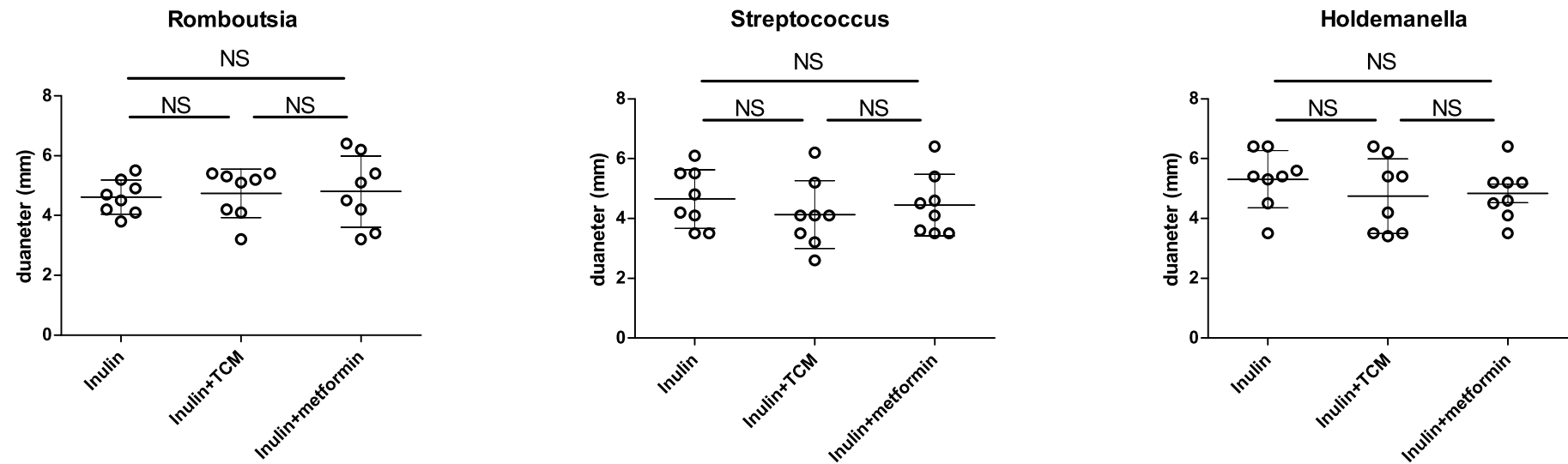

Supplement: Supplementary 4 — Supplementary Figure 2: In vitro assay exhibited that specific bacterium was not altered. (A) Microplate assay showed that Romboutsia, Streptococcus, and Holdemanella were not altered by different treatments. Data represents similar results acquired from 5 repeated experiments. NS: no significant. Each group contained 8 samples. (B) Agar well diffusion assay showed Romboutsia, Streptococcus, and Holdemanella were not altered by different treatments. Data represents similar results acquired from 5 repeated experiments. NS: no significant. Each group contained 8 samples. [file 2078520.f4.pdf]
